# Aromatherapy and Aromatic Plants for the Treatment of Behavioural and Psychological Symptoms of Dementia in Patients with Alzheimer's Disease: Clinical Evidence and Possible Mechanisms

**DOI:** 10.1155/2017/9416305

**Published:** 2017-03-30

**Authors:** Damiana Scuteri, Luigi Antonio Morrone, Laura Rombolà, Pina Rosa Avato, Anna Rita Bilia, Maria Tiziana Corasaniti, Shinobu Sakurada, Tsukasa Sakurada, Giacinto Bagetta

**Affiliations:** ^1^Department of Pharmacy, Health Science and Nutrition, University of Calabria, 87036 Rende, Italy; ^2^Department of Pharmacy and Drug Science, University of Bari “Aldo Moro”, 70125 Bari, Italy; ^3^Department of Chemistry, University of Florence, Sesto Fiorentino, 50019 Florence, Italy; ^4^Department of Health Sciences, University “Magna Græcia” of Catanzaro, 88100 Catanzaro, Italy; ^5^First Department of Pharmacology, Daiichi College of Pharmaceutical Sciences, Fukuoka, Japan; ^6^Department of Physiology and Anatomy, Tohoku Pharmaceutical University, Sendai, Japan

## Abstract

The treatment of agitation and aggression, typical Behavioural and Psychological Symptoms of Dementia (BPSDs) of Alzheimer's Disease (AD), is one of the most complicated aspects of handling patients suffering from dementia. Currently, the management of these symptoms often associated with an increased pain perception, which notably reduces the patients' quality of life (QoL), relies on the employment of antipsychotic drugs. Unfortunately, the use of these pharmacological agents has some limits: in the long term, they do not result in being equally effective as in the first weeks of treatment and they present important side effects. Therefore, there is growing interest, supported by clinical evidence, in aromatherapy for the control of agitation, aggression, and psychotic symptoms. Some molecular mechanisms have been proposed to explain the behavioural effects of essential oils, as the whole phytocomplex or the single components, but important basic research effort is still needed. For this reason, rigorous preclinical studies are necessary in order to understand the pharmacological basis of aromatherapy in the treatment of BPSDs and to widen the cluster of effective essential oils in pharmacotherapeutic practice.

## 1. Introduction: Characterization of Alzheimer's Disease

Alzheimer's Disease (AD), originally described by Alzheimer in 1907 [1], is the most common cause of dementia in the elderly: 35 million people all over the world are affected by dementia [2] and, according to the survey made by the World Health Organization (WHO) in 2012, 54% of all the cases of dementia are AD-related. These data account for the social burden of AD worldwide. Other forms of dementia are Lewy body dementia, frontotemporal dementia, and vascular dementia. AD is a progressive neurodegenerative disease characterized by cognitive and noncognitive dysfunctions [3]. In particular, the WHO describes dementia as a clinical syndrome due to disease of the brain, usually of a progressive nature, which leads to disturbances of multiple higher cortical functions, including memory, thinking, orientation, comprehension, calculation, learning capacity, language, and judgment. The cognitive deficits include memory impairment, aphasia, apraxia, agnosia, and disturbances in executive functioning [4, 5]. Although these are the fundamental goal in the long term, the most frequent issue for people with AD remains the management of Behavioural and Psychological Symptoms of Dementia (BPSDs) [6] and pain [7]. The pathogenesis of AD is explained by the amyloid hypothesis, according to which deposition and accumulation of amyloid *β*-peptide (A*β*) are responsible for the neurodegeneration; thus the reduction of A*β* plaques should produce clinical improvement [8]. Other neuropathological markers of AD are the neurofibrillary tangles (NFTs), composed of hyperphosphorylated* tau* proteins. Available drugs for the pharmacological treatment of AD are acetylcholinesterase inhibitors: Donepezil (Aricept, 1996), Rivastigmine (Exelon, 1998), and Galantamine (Razadyne, 2001). A more recently approved drug by Food and Drug Administration (FDA) is effective in AD-induced cognitive impairment, that is, the noncompetitive N-methyl-D-aspartate- (NMDA-) receptor antagonist Memantine (Namenda, 2003). New strategies targeting A*β* have been tested in order to delay AD progression, but small molecules and immunotherapy both have failed [9]. For this purpose, novel biological drugs have been studied as disease-modifying agents for AD. In particular, two placebo-controlled multicenter, double-blind phase 3 studies on mild-to-moderate AD, EXPEDITION 1 and EXPEDITION 2, unraveled that Solanezumab, a humanized antiamyloid monoclonal antibody, which binds soluble A*β*, did not show significant results concerned with the primary outcomes, failed to improve cognition, and did not enhance functional ability (funded by Eli Lilly; EXPEDITION 1 and EXPEDITION 2 ClinicalTrials.gov numbers NCT00905372 and NCT00904683) [10]. Also Bapineuzumab, a humanized anti-amyloid-beta monoclonal antibody, was studied in two double-blind, randomized, placebo-controlled phase 3 trials on mild-to-moderate AD; the results demonstrated that Bapineuzumab did not produce any improvements of the set clinical outcomes (funded by Janssen Alzheimer Immunotherapy and Pfizer; Bapineuzumab 301 and 302 ClinicalTrials.gov numbers NCT00575055 and NCT00574132 and EudraCT number 2009-012748-17.) [11]. Interestingly, Aducanumab (BIIB037), a human monoclonal antibody able to bind A*β* aggregates (soluble and insoluble form), was tested in a double-blind, placebo-controlled phase 1b randomized trial (PRIME; ClinicalTrials.gov identifier NCT01677572) involving patients showing prodromal or mild AD; Aducanumab administration resulted in a reduction of brain A*β* plaques in a dose- and time-dependent manner, as shown in Florbetapir PET imaging [12]. Moreover, Aducanumab trial demonstrated an improvement of the Mini Mental State Examination (MMSE) at one year, mainly at the doses of 3 and 10 mg/Kg and of the Clinical Dementia Rating-Sum of Boxes (CDR-SB) score at one year [12]. Therefore, the studies on Aducanumab support the amyloid hypothesis and demonstrate that the use of Aducanumab could be an interesting disease-modifying approach for AD treatment in the still ongoing long-term extension (LTE) phase of the PRIME and phase 3 studies [12].

## 2. Strategy for the Management of BPSDs

Dementia is widespread among patients hosts of nursing care homes, since up to 80% of them suffer from it [13]. Lots of patients affected by dementia, in particular 40–60% of the residents in care homes [14], develop BPSDs. These symptoms include agitation, aggression, psychotic manifestations with consequent stress, increased pain perception, and decreased quality of life (QoL). In order to establish the most suitable treatment for each individual condition, a rigorous evaluation of the symptoms and assessment of pain is necessary; the latter is made much more complicated in AD patients due to their difficulty to convey what they perceive. The atypical antipsychotic drugs have replaced the typical ones in the treatment of BPSDs because of the improved safety shown in the treatment of schizophrenic patients; indeed, the atypical antipsychotics (Risperidone, Olanzapine, Aripiprazole, and Quetiapine) were developed for schizophrenia therapy [15]. Among these drugs, Risperidone is the safest antipsychotic drug for short-term therapy of BPSDs in patients with dementia, even if it needs an accurate review of the treatment [15]. Furthermore, since chronic pain affects mainly the population of the elderly, it often accompanies patients suffering from dementia, determining a reduction of the QoL almost as remarkable as that provoked by memory loss. Pain takes on a pivotal importance in demented patients, because it plays a fundamental role in the development of the BPSDs, in particular of agitation and aggression [16]. In AD pain perception is impaired because of neuropathological changes of the pain systems, in particular in the locus coeruleus [17] and in the periaqueductal gray [18]. For this reason, the effect of pain treatment on patients' agitation has been tested. During a multicenter cluster randomized controlled trial (ClinicalTrials.gov NCT01021696 and Norwegian Medicines Agency EudraCTnr 2008-007490-20) [19], carried out in Norway from October 2009 to June 2010 in 60 nursing homes, 65-year-old or older demented patients affected by BPSDs (apart from the patients belonging to the control group that were treated as they usually were) received different analgesic drugs according to a standardized stepwise protocol: oral paracetamol, oral morphine, buprenorphine transdermal patch, or oral pregabalin. The primary outcome was agitation, assessed through the Cohen-Mansfield agitation inventory, while the secondary outcomes were aggression, pain, cognition, and daily activities. The results demonstrated a significant average reduction in agitation of 17% in the intervention group compared to the control group; the former presented an increased agitation score when painkillers were withdrawn [19]. Therefore, this study supports the importance of an adequate evaluation and treatment of pain for the management of agitation and aggression in patients suffering from dementia. Indeed, an appropriate treatment strategy for BPSDs needs a complex evaluation of the several multiple symptoms manifested by the patients, so that pharmacological agents are not administered if not or until necessary.

## 3. The Antipsychotics

The typical antipsychotics have been the first off-label pharmacological treatment of BPSDs. In order to study the effect of haloperidol in agitated demented patients, a search was made on databases such as MEDLINE, EMBASE, PsycInfo, and CINAHL: five randomized, placebo-controlled trials were included. The results yielded by the completed four randomized controlled trials (RCTs) have proven evidence for the effectiveness of haloperidol on aggression, when compared to placebo, but not on other manifestations of agitation [20, 21]. The relative effectiveness of these agents was counterbalanced by their serious side effects especially about the cardiac sphere and the parkinsonism. Indeed, a study on 495 psychiatric patients (with 101 healthy controls) demonstrated that these antipsychotic agents were predictors of QTc lengthening in a dose-related manner [22]. Thus, soon the novel atypical antipsychotics like Risperidone, Olanzapine, and Quetiapine took the place of the older typical agents in the management of aggression and agitation in dementia, thanks to their improved tolerability. Part of the amount of data collected in placebo-controlled trials on atypical antipsychotic drugs in AD is not available; at variance with the latter, all the results generated through meta-analyses on Risperidone and Aripiprazole are available [21]. In particular, these drugs result effective in controlling aggression mainly in a treatment period of 6–12 weeks, with larger evidence base for Risperidone [21, 23, 24]. Unfortunately, the randomized controlled trials lasting for a longer period have not highlighted efficacy of atypical antipsychotics in the long-term and, even when some effectiveness occurred, the incidence of side effects made it irrelevant in terms of cost/effectiveness [21, 25, 26]. For instance, a randomized double-blind placebo-controlled trial, conducted on 93 patients, guests of care facilities in Newcastle and who are affected by AD or dementia, showed that Quetiapine did not produce significant improvements in agitation score measured with Cohen-Mansfield agitation inventory but was associated with a worse decline of cognition assessed by severe impairment battery at 6 and 26 weeks [25]. Another double-blind placebo-controlled trial (ClinicalTrials.gov number NCT00015548) made on AD outpatients evaluated the effects of Olanzapine, Quetiapine, and Risperidone with respect to placebo [26]. The primary outcome of this trial, which made part of the National Institute of Mental Health (NIMH) Clinical Antipsychotic Trials of Intervention Effectiveness, was the time until discontinuation of treatment for any reason in phase 1: the median time to discontinuation of treatment for any reason resulted in being 5–8 weeks, without any significant differences among the three atypical antipsychotics and between them and the placebo [26]. Moreover, the time to discontinuation of treatment for intolerance to the drug, adverse effects, or death was better for the placebo group [26]. Some of the most important adverse events caused by atypical antipsychotics are drowsiness, extrapyramidal symptoms, and, more importantly, cerebrovascular accidents: according to the placebo-controlled trials, for Risperidone this possibility is 3-fold higher than placebo [21]. Another important matter to be dealt with is the cognitive decline: the use of atypical antipsychotic agents over 12 weeks is associated with an accelerated cognitive decline assessed by MMSE [24]. The FDA analyzed results of placebo-controlled trials and outlined a 1,6-1,7-fold increased mortality in patients subjected to atypical antipsychotics rather than placebo over 12 weeks [21, 27]. Moreover, the mortality at 12 months was evaluated in a randomized placebo-controlled, parallel, two-group treatment discontinuation trial [28]: the results highlighted increased mortality both within the 12 months' trial duration and during the 54 months of follow-up, thus outlining the persistence of the risk of mortality increase with the long-term treatment with antipsychotic drugs. By contrast, another study to assess the antipsychotic-associated long-term mortality was conducted throughout a 75-month follow-up period in 4 Norwegian counties, but it did not show an increased mortality [29].

## 4. Complementary Approach through Aromatherapy: Clinical Evidence and Possible Pharmacological Mechanisms

**Figure 1:**
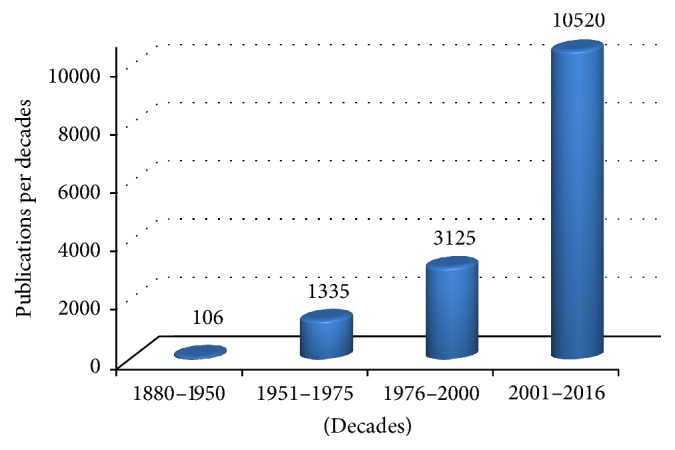
PubMed search on essential oils. An exponential growth of scientific production about essential oils is displayed in the figure from the 80s to 2016.

Since the atypical antipsychotics should be used only in short-term treatment and not over 12 weeks, there has been growing interest in the use of aromatherapy for BPSDs handling over the last years. Aromatherapy is a specialized segment of phytotherapy that uses essential oils, extracted from the different organs of aromatic plants, more often administered via inhalation or topical application and massage for several, minor, clinical uses [30]. A PubMed search, conducted using “Essential oils” as key word, provided the graph shown in Figure 1.

The remarkable increase of the scientific production dealing with essential oils, occurring from the 80s to 2016, accounts for the intensity of the phenomenon “aromatherapy.” Indeed, during the second half of the past century, since the first article published by Wood HC and Reichut ET in 1880 on the prestigious Journal of Physiology and entitled “Note on the Action upon the Circulation of Certain Volatile Oils,” the interest in aromatherapy for the treatment of several disorders such as anxiety, mood disorders, and certain forms of pain registered a great growth [30]. Furthermore, aromatherapy has provided the best evidence, together with psychological treatment, for the management of agitation in dementia [21]. In particular, the essential oils of two species of Lamiaceae family,* Melissa officinalis* L. (lemon balm) and* Lavandula officinalis *L. (lavender), are the most used aromatherapeutic treatments for BPSDs in dementia [31–33].* Melissa officinalis* L. (Lemon balm) belongs to the Lamiaceae family and is a traditional medicinal plant native of East Mediterranean region and West Asia and widespread in Teheran, where it is named* Badranjbooye* [34].

A placebo-controlled trial, conducted on patients affected by severe dementia guests of care facilities in the UK, reported the effect of* Melissa officinalis (M. officinalis) *essential oil, applied as massage twice a day for 4 weeks, on agitation measured by the Cohen-Mansfield agitation inventory (CMAI) [31]: seventy-one out of the seventy-two participants completed the trial and results demonstrated an improvement of agitation without the occurrence of significant side effects. The efficacy of lemon balm hydroalcoholic extracts rather than the essential oil is also well documented. In a study (a parallel group, double-blind, randomized, placebo-controlled trial), involving aged patients (from 65 to 80 years of age) suffering from mild-moderate AD, 60 drops/day of lemon balm extract were administered. Lemon balm exerted positive effects both on cognition, as measured through the 11-item cognitive subscale of the Alzheimer's Disease Assessment Scale (ADAS-cog) and the CDR-SB, and on agitation as side effect at 4 months [35]. The effectiveness of* M. officinalis* in AD could be explained by some cholinergic activities that have been detected in its extracts; this feature is shared also by* Salvia officinalis* (sage) and* Ginkgo biloba* [36, 37]. In a further study [38], the crude lemon balm hydroalcoholic extract has shown anticholinesterase activity. After fractionation, most of its fractions resulted in being more active than the whole extract. The constituents of the most active fractions are represented by* cis*- and* trans*-rosmarinic acid isomers and a rosmarinic acid derivative [38]. The high anticholinesterase activity and free radical scavenger properties of rosmarinic acid have been previously investigated by other authors [39]. Moreover, it is reported that a similar extract of* M. officinalis* contains compounds with acetylcholine receptor affinities being higher for the nicotinic subtype of receptors [40]. Therefore, the effects of* M. officinalis *could be due to the content of rosmarinic acid mainly [41–43] and also to the monoterpenoid constituents of the essential oil [41, 43]. Because of this hypothesis, a two-phase study was performed using lemon balm dried leaves [43]. Preliminarily, the inhibition of acetylcholinesterase (AChE) and nicotinic and muscarinic receptor-binding properties were evaluated for eight samples of dried leaves in human postmortem occipital cortex tissue. Subsequently, the sample of* M. officinalis* which resulted to have the highest cholinergic activity was administered to healthy young volunteers and the effects on cognition and mood were examined in a multiple-dose, multiple time-point, double-blind, placebo-controlled, balanced crossover study [43]. Twenty healthy young participants received single doses of 600, 1000, and 1600 mg of encapsulated dried leaf, or a matching placebo, at 7-day intervals. Cognitive performance and mood were assessed before dose and at 1, 3, and 6 h after dose, using the Cognitive Drug Research computerized assessment battery and the Bond–Lader visual analog scales, respectively. The obtained data supported the cholinergic receptor-binding properties of* M. officinalis* and the fact that it acts on mood and cognition in a dose- and time-dependent manner [43]. It is noteworthy that* M. officinalis* has shown some effects also on nociceptive behaviour in animal pain models and this could contribute to its effectiveness in the reduction of agitation. In fact, the mechanisms involved in the antinociceptive effects of a hydroalcoholic extract of* M. officinalis* and of rosmarinic acid have been recently investigated in mice [44]. In the formalin test, the extract provided a significant inhibition of both phases and inhibited in a dose-dependent manner the glutamate-induced pain. The antinociceptive effect elicited by this extract in the glutamate test was significantly attenuated by intraperitoneal (i.p.) treatment of mice with atropine, mecamylamine, or L-arginine but not with naloxone or D-arginine [44], thus supporting the involvement of the cholinergic system and of the L-arginine-nitric oxide pathway. In addition, the rosmarinic acid contained in this extract appeared to contribute to the antinociceptive features of* M. officinalis* extract [44].  Table 1 summarizes the main studies supporting the use of* Melissa officinalis* in aromatherapy.

Lavender belongs to the family Labiatae (Lamiaceae). Lavender essential oil is obtained by distillation of diverse members of the genus* Lavandula* and it has been used for centuries. Some of the enumerated most commonly used species are* L. officinalis (syn. L. angustifolia)*,* L. latifolia, *and* L. stoechas*.* L. angustifolia* has been used for centuries in folk medicine to treat anxiety and agitation: this is the reason of the growing investigation of lavender aromatherapy for the control of BPSDs. Lavender essential oil is composed of over 100 constituents, among which the principal are linalool (51%), linalyl acetate (35%), *α*-pinene, limonene, 1,8-cineole, cis- and trans-ocimene, 3-octanone, camphor, caryophyllene, terpinen-4-olandlavendulyl acetate, and cineole [45]. According to the existing literature, lavender essential oil is able to inhibit glutamate and GABA receptor binding [46–48] and to improve behavioural conflict between mice [48, 49]. Furthermore, lavender has been shown to lower plasma cortisol levels [48, 50, 51] and reduce the need for analgesia during the postoperative period in humans [48, 52]; a possible action of lavender essential oil on tryptophan has also been hypothesized [53, 54]. Lavender oil revealed also an interesting analgesic activity relevant after inhalation mainly, at doses devoid of sedative side effects [55]. In the hot plate test the oil inhalation induced an analgesic activity which was inhibited by naloxone, atropine, and mecamylamine before treatment, thus supporting the involvement of both opioidergic and cholinergic pathways [55]. A placebo-controlled trial including 15 demented patients affected by agitation, according to the Pittsburgh Agitation Scale (PAS), reported some effectiveness of 2% lavender oil aromatherapy stream [32]. The efficacy of aromatherapy could be partly due to the terpenes content of the used essential oils, since these molecules undergo quick lung absorption and are able to cross the blood-brain barrier [33]. A crossover randomized trial conducted on seventy Chinese aged demented patients suggested the efficacy of lavender administered by inhalatory route as an adjunctive therapy for the management of agitation [56]. Moreover, the effect of aromatherapy performed using more than one single essential oil on dementia and AD was tested. A group of 28 demented old patients, 17 of whom suffering from AD, was exposed to the aroma of 0.04 mL lemon and 0.08 mL rosemary essential oil in the morning and to the aroma of 0.08 mL lavender and 0.04 mL orange essential oils in the evening [57]. The rationale underneath this scheme relies on the possibility that the mixed lemon and rosemary aromas activate the sympathetic nervous system in order to improve concentration and memory in the morning and the mixed lavender and orange aromas activate the parasympathetic nervous system, thus making the patients quiet in the evening [57]. The results obtained so far support the clinical use of aromatherapy in AD patients [57].  Table 2 summarizes the main studies supporting the use of* Lavandula officinalis* in aromatherapy.

## 5. Conclusions and Future Perspectives

Dementia, of which AD represents the most prevalent etiological factor, is a very complex social problem and it has an even more remarkable burden in a future view, since it is expected to affect more than 115 million people all over the world by 2050 [58]. Apart from the urgent need of disease-modifying drugs able to delay the progression of the neurocognitive decline, another important aspect to deal with is the management of aggression, agitation, and psychotic symptoms, clustered under the acronym BPSDs, which contribute to reduce the QoL and, in particular, the health-related quality of life (HRQL) of patients suffering from dementia. The pharmacological treatment of BPSDs is represented by the atypical antipsychotics (Risperidone, Olanzapine, Aripiprazole, and Quetiapine), among which Risperidone is the safest drug for short-term therapy, with necessary review of the treatment [15]. The employed treatments, especially for BPSDs, affect the HRQL; the influence of person-centred care (PCC), antipsychotic review, social interaction, and exercise interventions on HRQL was studied in the Improving Wellbeing and Health for People with Dementia (WHELD) factorial cluster RCT in nursing home residents [59]. The obtained results support the importance of an adequate review of the antipsychotics use and of the use of evidence-based nonpharmacological approaches as combination therapy [59]. The atypical antipsychotic agents for the management of BPSDs in patients suffering from dementia should be used in the short term (6–12 weeks), since they result in being safer and effective in this treatment option (although almost exclusively on aggression rather than on agitation), under rigorous review and in combination with nonpharmacological interventions. Among the alternative treatments, aromatherapy has provided substantial evidence for agitation handling in AD [21]. The mechanism of action of constituents of aromatic plants has yet to be discovered. Detailed information on psychological effects of aromatic plants, their essential oils, and hydroalcoholic extracts has been reported. The aromatic molecules bind to olfactory epithelium acceptors that are specific for each different smell. The olfactory nerve system is responsible for the transmission of this stimulus to hippocampus, limbic system, and amygdala and then to the hypothalamus with consequent release of neuromediators [57]. The involvement of hippocampus and amygdala in the cognitive impairment characterizing dementia and the presence of neurofibrillary tangles (NFTs) in the entorhinal cortex, already in the early stages of AD [57, 60, 61], suggest an interesting link between olfaction and AD, furtherly confirmed by the dysfunctional olfaction by which demented patients are often affected [57]. It has been hypothesized that aromatherapy may promote neurogenesis in dentate gyrus of hippocampus [57, 62]. Accordingly, systemic absorption (following direct inhalation or inhalation after topical application) and distribution of pharmacologically active components of the phytocomplex are needed for aromatherapy to control BPSDs, and this may minimize the role of the psychological action [30]. The most used aromatherapeutic treatments for BPSDs in dementia are represented by* Melissa officinalis* and* Lavandula officinalis *[31–33]. Both of them have been used in traditional medicine for centuries: for example, the documented historical uses of* M. officinalis *date back to the “Materia Medica” in approximately 50–80 BC [43]. In addition, essential oils from both plants are included in the European Pharmacopoeia. Another fundamental facet is the increase of pain states in demented patients that are particularly related to agitation and aggression [16]. There is promising evidence for the effectiveness of aromatherapy for managing chronic pain: it was demonstrated that the intraplantar administration of Bergamot Essential Oil (BEO), a citrus fruit belonging to the Rutaceae family, significantly attenuates capsaicin-induced nociceptive behaviour [63] and both the first and the second phase of formalin-induced nocifensive response, thus confirming the previous observation that BEO reduces mechanical allodynia in neuropathic pain models [64–66]. Therefore, more rigorous RCTs assessing the effectiveness of aromatherapy on BPSDs, with recruitment of larger samples of patients and longer duration, are needed [21]. Additional basic research effort is necessary to understand the pharmacological mechanisms underlying aromatherapy. Finally, it is fundamental to carry out pharmacovigilance for ensuring a correct and safe application of aromatherapy.

## Figures and Tables

**Table 1:** Main studies supporting the use of *Melissa officinalis* in aromatherapy.

Study	Main characteristics
Ballard et al., 2002.	Placebo-controlled trial, conducted on seventy-two demented resident in care facilities in the UK (seventy-one out of whom completed the trial), demonstrating that *M. officinalis * essential oil, applied as massage twice a day for 4 weeks, produced an improvement of agitation (according to the CMAI), without the occurrence of significant side effects.

Akhondzadeh et al., 2003.	Parallel group, double-blind, randomized, placebo-controlled trial, involving aged patients (from 65 to 80 years of age) suffering from mild-moderate AD, who were given 60 drops/day of lemon balm extract. Lemon balm exerted positive effects both on cognition (according to the ADAS-cog and the CDR-SB) and on agitation as side effect at 4 months.

Dastmalchi et al., 2009.	Fractionation of the crude lemon balm hydroalcoholic extract, demonstrating anticholinesterase activity of most of the fractions, that resulted in being more active than the whole extract. The constituents of the most active fractions are *cis*- and *trans*-rosmarinic acid isomers and a rosmarinic acid derivative.

Kennedy et al., 2003.	Two-phase study: preliminary evaluation of the AChE inhibition and of nicotinic and muscarinic receptor-binding properties for eight samples of lemon balm dried leaves in human postmortem occipital cortex tissue and subsequent administration to 20 healthy young participants of the sample that resulted to have the highest cholinergic activity. The effects on cognition and mood were examined in a multiple-dose, multiple time-point, double-blind, placebo-controlled, balanced crossover study. The obtained data supported the cholinergic receptor-binding properties of *M. officinalis* and the fact that it acts on mood and cognition in a dose- and time-dependent manner.

Guginski et al., 2009.	Study performed using the formalin test in mice, in which the *M. officinalis* extract provided a significant inhibition of both phases and inhibited in a dose-dependent manner the glutamate-induced pain. The antinociceptive effect elicited by this extract in the glutamate test was significantly attenuated by i.p. administered atropine, mecamylamine, or L-arginine but not naloxone or D-arginine, thus supporting the involvement of the cholinergic system and of the L-arginine-nitric oxide pathway. The rosmarinic acid contained in this extract appeared to contribute to its antinociceptive features.

**Table 2:** Studies supporting the use of *Lavandula officinalis* in aromatherapy.

Study	Main characteristics
Barocelli et al., 2004.	In the hot plate test the lavender oil inhalation induced analgesic activity, which was inhibited by naloxone, atropine, and mecamylamine before treatment, thus supporting the involvement of both opioidergic and cholinergic pathways.

Holmes et al., 2002.	A placebo-controlled trial including 15 demented patients affected with agitation, as assessed by the PAS, which reported some effectiveness of 2% lavender oil aromatherapy stream.

Lin et al., 2007.	A cross-over randomized trial, conducted on 70 Chinese aged demented patients, which suggested the efficacy of lavender administered by inhalatory route as adjunctive therapy for the management of agitation.

Jimbo et al., 2009.	A group of 28 demented old patients, 17 of whom suffering from AD, was exposed to the aroma of 0.04 mL lemon and 0.08 mL rosemary essential oil in the morning and to the aroma of 0.08 mL lavender and 0.04 mL orange essential oils in the evening, in order to improve concentration and memory in the morning and to make the patients quiet in the evening. The results obtained so far support the clinical use of aromatherapy in AD patients, also when performed using more than one single essential oil.

## References

[1] Alzheimer A., Stelzmann R. A., Schnitzlein H. N., Murtagh F. R. (1995). An English translation of Alzheimer's 1907 paper, ‘Uber eine eigenartige Erkankung der Hirnrinde’. *Clinical Anatomy*.

[2] Ballard C., Orrell M., Zhong S. Y. (2016). Impact of antipsychotic review and nonpharmacological interventionon antipsychotic use, neuropsychiatric symptoms, and mortality in people with dementia living in nursing homes: a factorial cluster-randomized controlled trial by the well-being and health for people with dementia (WHELD) program. *American Journal of Psychiatry*.

[3] Barage S. H., Sonawane K. D. (2015). Amyloid cascade hypothesis: pathogenesis and therapeutic strategies in Alzheimer's disease. *Neuropeptides*.

[4] American Psychiatric Association (1987). *Diagnostic and Statistical Manual of Mental Disorders, Third Edition-Revised (DSM-III-R)*.

[5] American Psychiatric Association (1994). *Diagnostic and Statistical Manual of Mental Disorders*.

[6] Ballard C. G., Gauthier S., Cummings J. L. (2009). Management of agitation and aggression associated with Alzheimer disease. *Nature Reviews Neurology*.

[7] Achterberg W., Pieper M. J., van Dalen-Kok A. H. (2013). Pain management in patients with dementia. *Clinical Interventions in Aging*.

[8] Hardy J. A., Higgins G. A. (1992). Alzheimer's disease: the amyloid cascade hypothesis. *Science*.

[9] Cummings J. L., Morstorf T., Zhong K. (2014). Alzheimer's disease drug-development pipeline: few candidates, frequent failures. *Alzheimer's Research and Therapy*.

[10] Doody R. S., Thomas R. G., Farlow M. (2014). Phase 3 trials of solanezumab for mild-to-moderate Alzheimer's disease. *The New England Journal of Medicine*.

[11] Salloway S., Sperling R., Fox N. C. (2014). Two phase 3 trials of Bapineuzumab in mild-to-moderate Alzheimer's disease. *The New England Journal of Medicine*.

[12] Sevigny J., Chiao P., Bussière T. (2016). The antibody aducanumab reduces A*β* plaques in Alzheimer’s disease. *Nature*.

[13] Corbett A., Nunez K., Thomas A. (2013). Coping with dementia in care homes. *Maturitas*.

[14] Margallo-Lana M., Swann A., O'Brien J. (2001). Prevalence and pharmacological management of behavioural and psychological symptoms amongst dementia sufferers living in care environments. *International Journal of Geriatric Psychiatry*.

[15] Ballard C., Corbett A. (2013). Agitation and aggression in people with Alzheimer's disease. *Current Opinion in Psychiatry*.

[16] Corbett A., Husebo B., Malcangio M. (2012). Assessment and treatment of pain in people with dementia. *Nature Reviews Neurology*.

[17] Zarow C., Lyness S. A., Mortimer J. A., Chui H. C. (2003). Neuronal loss is greater in the locus coeruleus than nucleus basalis and substantia nigra in Alzheimer and Parkinson diseases. *Archives of Neurology*.

[18] Parvizi J., Van Hoesen G. W., Damasio A. (2000). Selective pathological changes of the periaqueductal gray matter in Alzheimer's disease. *Annals of Neurology*.

[19] Husebo B. S., Ballard C., Sandvik R., Nilsen O. B., Aarsland D. (2011). Efficacy of treating pain to reduce behavioural disturbances in residents of nursing homes with dementia: cluster randomised clinical trial. *BMJ*.

[20] Lonergan E., Luxenberg J., Colford J. (2002). Haloperidol for agitation in dementia. *The Cochrane Database of Systematic Reviews*.

[21] Ballard C. G., Gauthier S., Cummings J. L. (2009). Management of agitation and aggression associated with alzheimer disease. *Nature Reviews Neurology*.

[22] Reilly J. G., Ayis S. A., Ferrier I. N., Jones S. J., Thomas S. H. L. (2000). QTc-interval abnormalities and psychotropic drug therapy in psychiatric patients. *The Lancet*.

[23] Ballard C., Howard R. (2006). Neuroleptic drugs in dementia: benefits and harm. *Nature Reviews Neuroscience*.

[24] Schneider L. S., Dagerman K., Insel P. S. (2006). Efficacy and adverse effects of atypical antipsychotics for dementia: meta-analysis of randomized, placebo-controlled trials. *The American Journal of Geriatric Psychiatry*.

[25] Ballard C., Margallo-Lana M., Juszczak E. (2005). Quetiapine and rivastigmine and cognitive decline in Alzheimer's disease: randomised double blind placebo controlled trial. *British Medical Journal*.

[26] Schneider L. S., Tariot P. N., Dagerman K. S. (2006). Effectiveness of atypical antipsychotic drugs in patients with Alzheimer's disease. *The New England Journal of Medicine*.

[27] FDA Public Health Advisory Deaths with antipsychotics in elderly patients with behavioral disturbances. https://www.fda.gov/Drugs/DrugSafety/ucm053171.htm.

[28] Ballard C., Hanney M. L., Theodoulou M. (2009). The dementia antipsychotic withdrawal trial (DART-AD): long-term follow-up of a randomised placebo-controlled trial. *The Lancet Neurology*.

[29] Selbæk G., Aarsland D., Ballard C. (2016). Antipsychotic drug use is not associated with long-term mortality risk in Norwegian nursing home patients. *Journal of the American Medical Directors Association*.

[30] Bagetta G., Cosentino M., Sakurada T. (2016). *Preface in Aromatherapy: Basic Mechanisms and Evidence Based Clinical Use*.

[31] Ballard C. G., O'Brien J. T., Reichelt K., Perry E. K. (2002). Aromatherapy as a safe and effective treatment for the management of agitation in severe dementia: the results of a double-blind, placebo-controlled trial with Melissa. *Journal of Clinical Psychiatry*.

[32] Holmes C., Hopkins V., Hensford C., MacLaughlin V., Wilkinson D., Rosenvinge H. (2002). Lavender oil as a treatment for agitated behaviour in severe dementia: a placebo controlled study. *International Journal of Geriatric Psychiatry*.

[33] Burns A., Byrne J., Ballard C., Holmes C. (2002). Sensory stimulation in dementia. *British Medical Journal*.

[34] Zarei A., Changizi Ashtiyani S., Taheri S., Rasekh F. (2014). Comparison between effects of different doses of Melissa officinalis and atorvastatin on the activity of liver enzymes in hypercholesterolemia rats. *Avicenna Journal of Phytomedicine*.

[35] Akhondzadeh S., Noroozian M., Mohammadi M., Ohadinia S., Jamshidi A. H., Khani M. (2003). *Melissa officinalis* extract in the treatment of patients with mild to moderate Alzheimer's disease: a double blind, randomised, placebo controlled trial. *Journal of Neurology, Neurosurgery, and Psychiatry*.

[36] Perry E. K., Pickering A. T., Wang W. W., Houghton P., Perry N. S. L. (1998). Medicinal plants and Alzheimer's disease: integrating ethnobotanical and contemporary scientific evidence. *Journal of Alternative and Complementary Medicine*.

[37] Perry E. K., Pickering A. T., Wang W. W., Houghton P. J., Perry N. S. L. (1999). Medicinal plants and Alzheimer's disease: from ethnobotany to phytotherapy. *Journal of Pharmacy and Pharmacology*.

[38] Dastmalchi K., Ollilainen V., Lackman P. (2009). Acetylcholinesterase inhibitory guided fractionation of *Melissa officinalis* L.. *Bioorganic and Medicinal Chemistry*.

[39] Petersen M., Simmonds M. S. J. (2003). Rosmarinic acid. *Phytochemistry*.

[40] Wake G., Court J., Pickering A., Lewis R., Wilkins R., Perry E. (2000). CNS acetylcholine receptor activity in European medicinal plants traditionally used to improve failing memory. *Journal of Ethnopharmacology*.

[41] Carnat A. P., Carnat A., Fraisse D., Lamaison J. L. (1998). The aromatic and polyphenolic composition of lemon balm (Melissa officinalis L. subsp. officinalis) tea. *Pharmaceutica Acta Helvetiae*.

[42] Hohmann J., Zupkó I., Rédei D. (1999). Protective effects of the aerial parts of Salvia officinalis, Melissa officinalis and Lavandula angustifolia and their constituents against enzyme- dependent and enzyme-independent lipid peroxidation. *Planta Medica*.

[43] Kennedy D. O., Wake G., Savelev S. (2003). Modulation of mood and cognitive performance following acute administration of single doses of *Melissa officinalis* (Lemon balm) with human CNS nicotinic and muscarinic receptor-binding properties. *Neuropsychopharmacology*.

[44] Guginski G., Luiz A. P., Silva M. D. (2009). Mechanisms involved in the antinociception caused by ethanolic extract obtained from the leaves of Melissa officinalis (lemon balm) in mice. *Pharmacology Biochemistry and Behavior*.

[45] Cavanagh H. M. A., Wilkinson J. M. (2002). Biological activities of lavender essential oil. *Phytotherapy Research*.

[46] Elisabetsky E., Marschner J., Onofre Souza D. (1995). Effects of linalool on glutamatergic system in the rat cerebral cortex. *Neurochemical Research*.

[47] Huang L., Abuhamdah S., Howes M.-J. R. (2008). Pharmacological profile of essential oils derived from *Lavandula angustifolia* and *Melissa officinalis* with anti-agitation properties: focus on ligand-gated channels. *Journal of Pharmacy and Pharmacology*.

[48] O'Connor D. W., Eppingstall B., Taffe J., Van Der Ploeg E. S. (2013). A randomized, controlled cross-over trial of dermally-applied lavender (Lavandula angustifolia) oil as a treatment of agitated behaviour in dementia. *BMC Complementary and Alternative Medicine*.

[49] Umezu T. (2000). Behavioral effects of plant-derived essential oils in the geller type conflict test in mice. *Japanese Journal of Pharmacology*.

[50] Shiina Y., Funabashi N., Lee K. (2008). Relaxation effects of lavender aromatherapy improve coronary flow velocity reserve in healthy men evaluated by transthoracic Doppler echocardiography. *International Journal of Cardiology*.

[51] Field T., Field T., Cullen C. (2008). Lavender bath oil reduces stress and crying and enhances sleep in very young infants. *Early Human Development*.

[52] Kim J. T., Ren C. J., Fielding G. A. (2007). Treatment with lavender aromatherapy in the post-anesthesia care unit reduces opioid requirements of morbidly obese patients undergoing laparoscopic adjustable gastric banding. *Obesity Surgery*.

[53] Zeilmann C. A., Dole E. J., Skipper B. J., McCabe M., Low Dog T., Rhyne R. L. (2003). Use of herbal medicine by elderly Hispanic and non-Hispanic white patients. *Pharmacotherapy*.

[54] Fu C.-Y., Moyle W., Cooke M. (2013). A randomised controlled trial of the use of aromatherapy and hand massage to reduce disruptive behaviour in people with dementia. *BMC Complementary and Alternative Medicine*.

[55] Barocelli E., Calcina F., Chiavarini M. (2004). Antinociceptive and gastroprotective effects of inhaled and orally administered *Lavandula hybrida Reverchon* ‘*grosso*’ essential oil. *Life Sciences*.

[56] Lin P. W.-K., Chan W.-C., Ng B. F.-L., Lam L. C.-W. (2007). Efficacy of aromatherapy (Lavandula angustifolia) as an intervention for agitated behaviours in Chinese older persons with dementia: a cross-over randomized trial. *International Journal of Geriatric Psychiatry*.

[57] Jimbo D., Kimura Y., Taniguchi M., Inoue M., Urakami K. (2009). Effect of aromatherapy on patients with Alzheimer's disease. *Psychogeriatrics*.

[58] World Health Organization and Alzheimer's Disease International (2012). *Dementia: A Public Health Priority*.

[59] Ballard C., Orrell M., Sun Y. (2016). Impact of antipsychotic review and nonpharmacological intervention on antipsychotic use, neuropsychiatric symptoms, and mortality in people with dementia living in nursing homes: a factorial cluster-randomized controlled trial by the Well-Being and Health for People With Dementia (WHELD) program. *The American Journal of Psychiatry*.

[60] Braak H., Braak E. (1991). Neuropathological stageing of Alzheimer-related changes. *Acta Neuropathologica*.

[61] Gold G., Bouras C., Kövari E. (2000). Clinical validity of Braak neuropathological staging in the oldest-old. *Acta Neuropathologica*.

[62] Eriksson P. S., Perfilieva E., Björk-Eriksson T. (1998). Neurogenesis in the adult human hippocampus. *Nature Medicine*.

[63] Sakurada T., Kuwahata H., Katsuyama S. (2009). Chapter 18 intraplantar injection of bergamot essential oil into the mouse hindpaw. Effects on capsaicin-induced nociceptive behaviors. *International Review of Neurobiology*.

[64] Bagetta G., Morrone L. A., Rombolà L. (2010). Neuropharmacology of the essential oil of bergamot. *Fitoterapia*.

[65] Kuwahata H., Komatsu T., Katsuyama S. (2013). Peripherally injected linalool and bergamot essential oil attenuate mechanical allodynia via inhibiting spinal ERK phosphorylation. *Pharmacology Biochemistry and Behavior*.

[66] Katsuyama S., Otowa A., Kamio S. (2015). Effect of plantar subcutaneous administration of bergamot essential oil and linalool on formalin-induced nociceptive behavior in mice. *Biomedical Research*.

